# Imbalances in Mobilization and Activation of Pro-Inflammatory and Vascular Reparative Bone Marrow-Derived Cells in Diabetic Retinopathy

**DOI:** 10.1371/journal.pone.0146829

**Published:** 2016-01-13

**Authors:** Harshini Chakravarthy, Eleni Beli, Svetlana Navitskaya, Sandra O’Reilly, Qi Wang, Nermin Kady, Chao Huang, Maria B. Grant, Julia V. Busik

**Affiliations:** 1 Department of Physiology, Michigan State University, East Lansing, Michigan, United States of America; 2 Department of Ophthalmology, Indiana University School of Medicine, Indianapolis, Indiana, United States of America; Children's Hospital Boston, UNITED STATES

## Abstract

Diabetic retinopathy is a sight-threatening complication of diabetes, affecting 65% of patients after 10 years of the disease. Diabetic metabolic insult leads to chronic low-grade inflammation, retinal endothelial cell loss and inadequate vascular repair. This is partly due to bone marrow (BM) pathology leading to increased activity of BM-derived pro-inflammatory monocytes and impaired function of BM-derived reparative circulating angiogenic cells (CACs). We propose that diabetes has a significant long-term effect on the nature and proportion of BM-derived cells that circulate in the blood, localize to the retina and home back to their BM niche. Using a streptozotocin mouse model of diabetic retinopathy with GFP BM-transplantation, we have demonstrated that BM-derived circulating pro-inflammatory monocytes are increased in diabetes while reparative CACs are trapped in the BM and spleen, with impaired release into circulation. Diabetes also alters activation of splenocytes and BM-derived dendritic cells in response to LPS stimulation. A majority of the BM-derived GFP cells that migrate to the retina express microglial markers, while others express endothelial, pericyte and Müller cell markers. Diabetes significantly increases infiltration of BM-derived microglia in an activated state, while reducing infiltration of BM-derived endothelial progenitor cells in the retina. Further, control CACs injected into the vitreous are very efficient at migrating back to their BM niche, whereas diabetic CACs have lost this ability, indicating that the *in vivo* homing efficiency of diabetic CACs is dramatically decreased. Moreover, diabetes causes a significant reduction in expression of specific integrins regulating CAC migration. Collectively, these findings indicate that BM pathology in diabetes could play a role in both increased pro-inflammatory state and inadequate vascular repair contributing to diabetic retinopathy.

## Introduction

DR is an important long-term complication of diabetes, affecting around 93 million people and is a leading cause of blindness among working adults worldwide [1]. The initial stages of DR are characterized by various clinical features including increased microvascular permeability, vessel leakage and appearance of microaneurysms [2]. Diabetic metabolic insult affects retinal vascular degeneration at several levels: First, by contributing to chronic retinal low-grade inflammation resulting in endothelial cell injury [3–6]; Second, by inadequate repair of the injured retinal capillaries by bone marrow (BM)-derived circulating angiogenic cells (CACs), which are exquisitely sensitive to the damaging diabetic milieu [7, 8]; finally, by activating monocytes [9] and further promoting a pro-inflammatory environment in the retina [10]. Retinal endothelial cell injury, activated monocytes and failed attempts by CACs to repair injured retinal capillaries collectively result in progression to the vasodegenerative stage of the disease [11–13].

Efficient release of CACs from the BM and spleen into circulation and extravasation into blood vessels in the tissues is a critical component of their surveillance and vascular repair function. We have previously shown that BM neuropathy precedes retinal vascular degeneration in DR, leading to trapping of diabetic progenitor cells in the BM, and affecting circadian release of these cells into circulation [7]. Homeostatic recirculation of cells back to the BM niche is an equally important aspect of their role in maintaining the BM progenitor microenvironment [14–16]. Chemokine gradients such as SDF-1, and up-regulation of specific receptors such as CXCR-4 on the CACs are believed to play crucial roles in regulating the process of homing and retention in niches [17, 18]. Expression of specific integrins such as α4β1, β2 and αvβ3 by CACs are major determinants of CAC adhesion to endothelial cells, homing and mobilization from the BM [19, 20]. However, the effect of diabetes on the ability of CACs to home from the tissues back to their BM niche has not been adequately studied.

Besides hosting the CACs, the BM is an important niche for several cells types such as stem cells, stromal supporting cells, myeloid and lymphoid precursors. Some of these cell types are recruited to the retina from the BM for retinal remodeling. The hematopoietic progenitors are also known to migrate from the BM to other niches such as peripheral blood and spleen [21, 22]. Interestingly, spleen acts as an important reservoir during CAC trafficking and as a storage site for lymphocytes, dendritic cells (DC) and monocyte populations [22, 23]. Leukocytes can be potentially activated by interaction with BM-derived DC, which secrete cytokines in response to immune stimulation and determine the nature of the leukocyte response during inflammation [24–26]. Aberrant activation of immune cells, as well as decreased mobilization of CACs may contribute to vascular complications in diabetes [23, 27–29].

The BM is also the source of myeloid-derived circulating monocytes, which contribute to DR-associated inflammation. We have previously demonstrated that diabetes induces a shift in hematopoiesis resulting in a reduction of reparative cells (CACs) and an increase in pro-inflammatory monocytes that are released into circulation [7, 30, 31]. Just like CAC dysfunction, immune cell imbalance and inflammation are critical participants in the pathogenic events associated with DR [10, 32]. Previously, we have shown that diabetes leads to increased accumulation of inflammatory monocytes in the retina [30]. It has been shown recently that pro-inflammatory BM-derived myeloid cells like monocytes play an important role in retinal endothelial cell death and capillary degeneration in diabetes [33]. However, the influence of diabetes on a range of other types of BM-derived cells, their migration to niches such as spleen and peripheral blood, and their association with retinal vasculature has not been explored in detail.

In this study, we propose that diabetes has a significant long-term effect on the nature and proportion of BM-derived cells that circulate in the blood, localize to the retina and home back to their BM niche. To test this hypothesis, we generated chimeric mice with long-term, stable reconstitution of their BM with GFP^+^ cells. After four months to allow for stable reconstitution of the BM, diabetes was induced by streptozotocin (STZ) injections and the retinas were analyzed for the type and number of BM-derived cells after 8 weeks of diabetes.

## Materials and Methods

### Mice

All procedures involving the animal models were approved by the Institutional Animal Care and Use Committee at Michigan State University. Male C57BL/6J and C57BL/6-Tg(CAG-EGFP) mice were purchased from Jackson Laboratory and made diabetic by injections of streptozotocin (STZ) dissolved in 0.5% sodium citrate buffer, with a daily dose of 65mg/Kg for five consecutive days. The control mice received sodium citrate buffer only. Mice with blood glucose greater than 13.8 mmol/L were considered diabetic. Starting 14 days after STZ injections, insulin injections (with a dose of 0–2 units/day) were administered to prevent acute weight loss, but allowing hyperglycemia in the range of 20 mmol/L blood glucose.

### Generation of chimeric mice

The C57BL/6-Tg(CAG-EGFP) transgenic donor strain was obtained from Jackson Laboratory. Chimeric mice were generated by irradiating recipient 8-weeks old C57BL/6 mice with 1100 rads followed by retroorbital injection of whole bone marrow (2 X 10^6^ cells) from donor C57BL/6 or GFP^+^ mice. After 120–130 days to allow stable hematopoietic reconstitution, we performed flow cytometry to enumerate GFP^+^ cells in the BM of chimeric mice. Diabetes was induced using STZ as described above.

### Isolation of hematopoietic progenitors/CACs from mice

Mice were euthanized and tibias and femurs were collected. The bones were flushed with ice-cold PBS and made into a single cell suspension. The cell pellet was treated with ammonium chloride solution (STEMCELL technologies) to remove contaminating red blood cells. The bone marrow cells were then enriched for mouse hematopoietic stem/progenitor cells using a lineage-negative selection kit (STEMCELL Technologies) followed by a Sca1 positive selection kit (STEMCELL Technologies) to obtain Lin^-^ Sca^+^ progenitor cells.

### Homing of CACs from retina

Lin^-^ Sca^+^ progenitor cells were obtained from GFP^+^ diabetic and control mice, as described above. The duration of diabetes was 9 months. The cells were maintained overnight in EGM-2 media with SingleQuot supplements and growth factors (Lonza) to allow them to recover from the isolation process. Cells were then washed with PBS, counted and 10,000 cells were injected intravitreously using a 33-gauge Hamilton syringe, into healthy wild type mice. After 7 days, the wild type mice were sacrificed, and their eyes removed.

### Tissue preparation and Immunohistochemistry

Eyes were pierced with a 30-gauge needle and fixed in freshly prepared 4% paraformaldehyde for 1 hour at room temperature. The eyes were then washed in three changes of PBS before dissection. Intact retinas were isolated and permeabilized overnight at 4°C in HEPES-buffered saline containing 0.1% Tween 20 and 1% bovine serum albumin. Vasculature was stained with rabbit anti-collagen IV (abcam) diluted 1:400 in PBS with 2% non-immune goat serum, incubating overnight at 4°C, followed by a change into PBS for 6–8 hours. Secondary antibody chicken anti-rabbit (Alexa Fluor 594, Invitrogen) diluted 1:1000 was used, followed by a final wash in PBS.

For characterization of vascular and perivascular GFP^+^ cells in chimeric mice, retinas were further stained with primary antibodies: endothelial cells using 1:400 diluted rabbit anti-collagen IV (abcam); astrocytes using 1:200 diluted rabbit anti-GFAP (Cell Signaling); microglial cells using 1: 100 diluted goat anti-Iba1 (Novus Biologicals); pericytes using 1:100 diluted rabbit anti-PDGFR-β (abcam); and Müller cells using 1:300 diluted rabbit anti-glutamine synthetase (Novus Biologicals). After overnight incubation at 4°C and three PBS washes, respective chicken anti-rabbit or anti-goat secondary antibodies (Alexa Fluor 594, Invitrogen) diluted 1:1000 was used, stained specimens incubated for an hour, followed by a final wash in PBS. Retinas were mounted flat with four to five radial incisions, and placed between glass coverslips with Fluoromount medium (Sigma).

### Tissue sectioning and Immunohistochemistry

Tissue samples previously fixed in Zinc Fixative (BD biosciences) were processed and vacuum infiltrated with paraffin on the ThermoFisher Excelsior tissue processor; followed by embedding with the ThermoFisher HistoCentre III embedding station. Once blocks were cooled, excess paraffin was removed from the edges, and they were placed on a Reichert Jung 2030 rotary microtome exposing the tissue sample. Then the blocks were cooled and finely sectioned at 4–5 microns. Sections were dried at a 56°C slide incubator to ensure adherence to the slides for 2–24 hours. Slides were then deparaffinized and rinsed in several changes of distilled water followed by Tris buffered saline pH 7.4. Sections were incubated with 10% normal blocking serum (chicken serum, Santa Cruz Biotechnology) in PBS for 20 minutes to suppress non-specific binding of IgG, and then washed with PBS. For localization of GFP^+^ cell types in the different layers of the retina, retinas were stained with primary antibodies for vascular and perivascular cells such as endothelial cells, astrocytes, microglial cells and Müller cells using antibodies and dilutions described above. Further, retinas were also stained for retinal neuronal cells such as amacrine cells using 1:200 diluted rabbit anti-tyrosine hydroxylase (Millipore), rod photoreceptors using 1:200 diluted rabbit anti-rhodopsin (Sigma) and ganglion cells using 1:50 diluted goat Brn-3a (Santa Cruz Biotechnology). After 1 hour incubation and three PBS washes, sections were incubated for an hour with Alexa Fluor 594-conjugated chicken secondary antibody against rabbit or goat (Invitrogen), diluted to 5 μg/ml, followed by a final wash in PBS. Retinal sections were mounted on coverslips with Fluoromount medium (Sigma).

### Sample processing and LPS treatment

Mice were euthanized and tibias and femurs were collected. The bones were flushed with ice-cold PBS and made into a single cell suspension. The cell pellet was treated with ammonium chloride solution (STEMCELL technologies) to remove red blood cells. For enrichment of dendritic cells, 1 million BM cells per well in 24-well plates were incubated at 37°C for 7 days in R10 medium (RPMI 1640 with 10% fetal bovine serum, 100 U/mL penicillin, 100 μg/mL streptomycin and 55 μM β-mercaptoethanol) supplemented with 10 ng/mL of GM-CSF (Peprotech). The culture medium was changed every 2 days by aspirating 50% of the medium and adding back fresh medium with supplements. Spleen was gently crushed, subjected to red blood cell lysis and then filtered through a 40μm nylon mesh. 1 million splenocytes per well in 24-well plates were also maintained in R10 medium for 7 days. The dendritic cell-enriched population from BM and splenocytes were stimulated with 10 ng/mL of lipopolysaccharide (LPS, Sigma) for 24 hours. Culture supernatant was collected and stored at -80°C. ELISA (eBiosciences) was performed to measure cytokine levels of IL-1β and TNF-α.

### Cell preparation and Flow cytometry

Single cell suspensions from bone marrow and spleen were obtained as described earlier. Blood was collected in heparinized tubes, and mononuclear cells were isolated using 1083 Histopaque (Sigma-Aldrich) according to manufacturer’s instructions. Eyes were collected, the retinas were isolated and disrupted mechanically by vigorous pipetting and digestion with 0.5 mg/ml collagenase D (Roche, Indianapolis, IN) and 750 U/ml DNase (Sigma) in HBSS for 15 min at 37 oC according to Kerr *et al* [34]. One million cells were stained with the appropriate antibodies on ice for 30 minutes according to standard cell surface staining protocol. The primary conjugated monoclonal antibodies that were used were purchased from BD biosciences or ebiosciences: PE-CD34 (RAM34), PerCPCy5.5 –Ly6A/E (D7), biotin- lineage (CD3e, CD45RA, GR1, CD11b, TER119), Alexa Fluor 700- CD45 (30-F11), APC-CD309 (Avas 12α1), PECy7-CD117 (2B8), streptavidin APC efluor780, PE-Tie2 (TEK4), PerCP efluor710-CD31 (390), APC Cy7-CD11b (M1/70), PerCPefluor 710-F4/80 (BM8), PE-Ly6G (1A8), PECy7-Ly6C (AL-21), APC-CD90.2 (53–2.1), FITC-CD61 (2C9.G3), PECy7-CD29 (HMb1-1), PE-CD49d (R1-2), FITC-CD18 (M18/2), PECy7-CD49f (GoH3), PE-CD51 (RMV-7). Dead cells were excluded using DAPI staining. Retinal endothelial cells were gated as CD45^-^ CD31^+^ and Tie2^+^ cells. Retina microglial cells were gated as CD45^-/dim^, CD11b^+^ cells. CACs in bone marrow, blood and spleen were defined as CD45^dim/+^, lineage^-^, CD34^+^ and CD309^+^ cells. Integrin expression (subunit β1:CD29, β2:CD18, β3: CD61, α4:CD49d, α6:CD49f and αv:CD51) was detected after gating on CACs. Data were acquired with a LSR II instrument (BD) with three lasers at 488, 405 and 640 at the Flow Cytometry Core at Michigan State University and data were analyzed with FlowJo software (Tree Star, Inc.).

### Data Collection and Analysis

Digital images of flat-mounted retinas were captured using an Olympus FluoView 1000 Laser Scanning confocal microscope. For imaging retinal sections, a Nikon TE2000 fluorescence microscope equipped with Photometrics CoolSNAP HQ2 camera was used. A minimum of three random fields was captured for each retina. Colocalization of green (for GFP^+^ cells) and red (stained vascular endothelium or other retinal cells) fluorescence was examined and area of fluorescence calculated using MetaMorph imaging system (Molecular Devices, Downingtown, PA).

### Statistical analyses

Data are presented as mean ± S.E.M. Results were analyzed for statistical significance by the Student’s t-test or one-way ANOVA followed by Tukey’s or Bonferroni’s post-hoc test (GraphPad Prism5, GraphPad Software, San Diego, CA), where appropriate.

## Results

### Characterization of BM-derived cells in control retina

**Fig 1:**
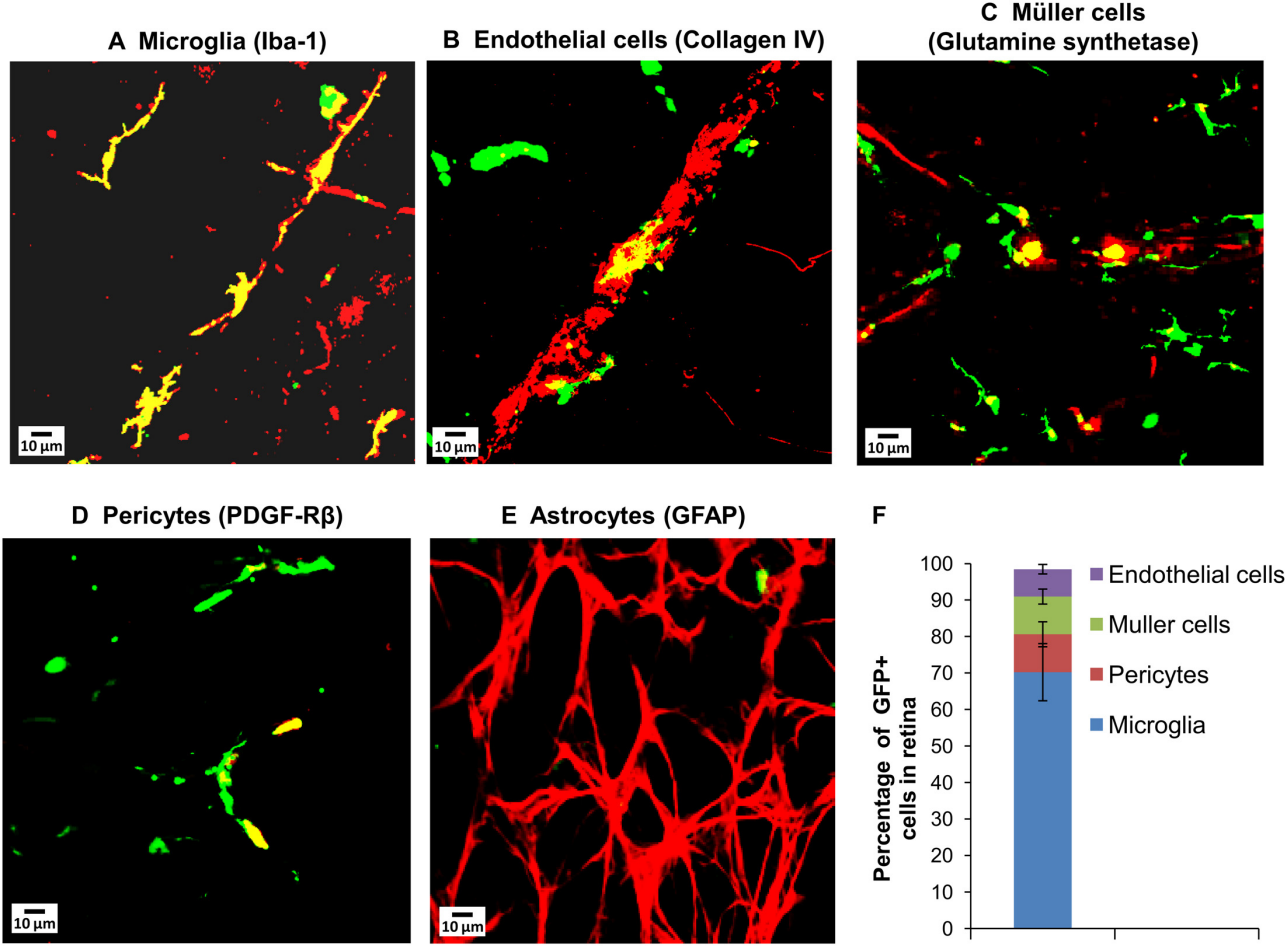
Characterization of BM-derived cells in retinas of GFP^+^ BM chimeras at 6 months after transplantation. GFP^+^ cells (green) colocalized with (A) microglial marker Iba-1 (red), (B) endothelial cell marker Collagen IV (red), (C) Müller cell marker Glutamine synthetase (red), (D) pericyte marker PDGFR-β (red) but not with (E) astrocyte marker GFAP (red). Colocalization of GFP^+^ cells with the respective markers was observed as yellow stain. Scale bars are 10 μm. (F) Percentages of GFP^+^ cells expressing markers for specific cell types, N = 4–5.

To track the movement of BM-derived cells into the retina, we created chimeric mice on a C57BL/6J background by transplanting with GFP^+^ age-matched BM at 8 weeks of age. 98% reconstitution of transplanted BM was confirmed by flow cytometry. 6 months after BM transplantation, characterization of the vasculature-associated GFP^+^ cells in retinas of chimeric mice by immunofluorescent staining of flat-mounted retinas indicated that BM-derived cells predominantly express markers of perivascular microglia, pericytes, Müller cells or vascular endothelial cells (Fig 1A–1D). A majority of the BM-derived cells infiltrating the retina expressed the pan-microglial marker, Iba-1 (Fig 1F). BM-derived astrocytes were not observed in the neural retina or associated with the retinal vasculature (Fig 1E).

**Fig 2:**
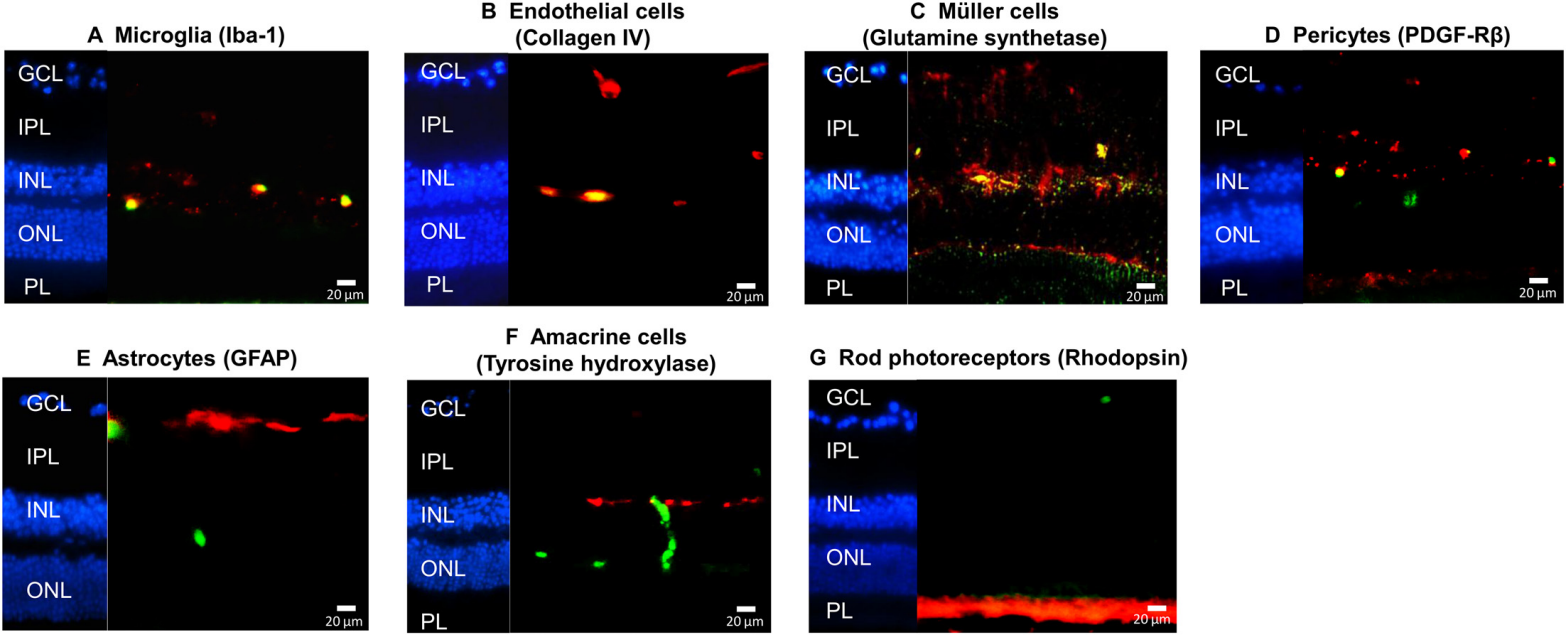
Characterization of BM-derived cells in retinal sections of GFP^+^ BM chimeras at 6 months after transplantation. Nuclei were counterstained with DAPI (blue). Overlays of confocal images of GFP^+^ cells (green) immunoreactive for (A) Iba1 (red) labeling microglia, (B) Collagen IV (red) labeling endothelial cells, (C) Glutamine synthetase (red) labeling Müller glia, (D) PDGF-Rβ (red) labeling pericytes. Colocalization of GFP^+^ cells with the respective markers was observed (yellow) in (A-D). (E) GFAP (red) labeling astrocytes, (F) Tyrosine hydroxylase (red) labeling amacrine cells, (G) Rhodopsin (red) labeling rod photoreceptors. No colocalization of GFP^+^ cells with these markers was observed in (E-G). Scale bars are 20 μm. N = 4–5.

To further analyze localization of the different BM-derived cell types within the retinal cell layers, immunohistochemical staining of retinal sections was performed. GFP^+^ cells in the retina localized predominantly to the ganglion cell layer (GCL), inner nuclear layer (INL), inner (IPL) and outer plexiform layers (OPL), where the retinal vasculature is located (Fig 2). The BM-derived cells in the retina were immunoreactive for Iba1 (labelling microglia, Fig 2A), collagen IV (labelling endothelial cells, Fig 2D), glutamine synthetase (labelling Müller cells, Fig 2C) and PDGF-Rβ (labelling pericytes, Fig 2B). However, GFP^+^ cells in the retinas were not recognized by antibodies against GFAP (labelling astrocytes, Fig 2E) and neuronal markers such as tyrosine hydroxylase (labelling amacrine cells, Fig 2F) and rhodopsin (labelling rod photoreceptors, Fig 2G). The observed fate of BM-derived cells in the retina is in agreement with a previous study characterizing BM-derived cells in the mouse retina [35].

**Fig 3:**
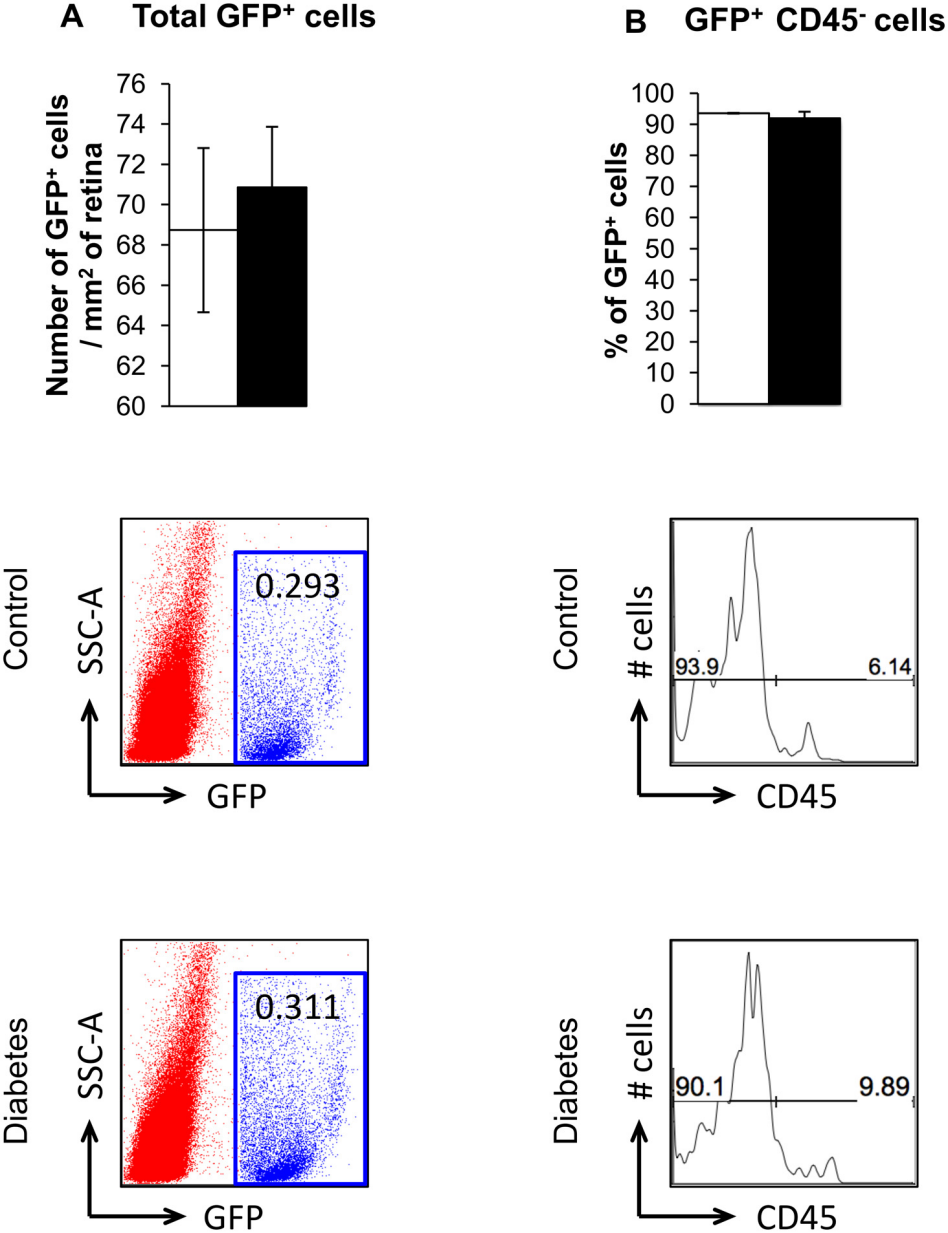
BM-derived cells in the retina of chimeric mice. (A) Number of BM-derived cells per mm^2^ area of control or diabetic retina. Representative flow charts of GFP^+^ cells in the retina shown below. (B) ~ 93% of GFP^+^ cells detected in the retina are CD45^-^ cells. Diabetes does not change the number of CD45^-^ cells in the retina. Representative flow charts gated on GFP^+^ cells of CD45^-^ and CD45^+^ cells shown below. N = 4.

Flow cytometry of GFP^+^ cells in retinas of chimeric mice was done to further confirm the immunohistochemical data. We observed that 93% of the GFP^+^ cells in the retina were CD45^dim^ or CD45^-^ cells (Fig 3B). The CD45 marker is expressed in high levels by all differentiated hematopoietic cells but not endothelial cells, [36, 37], and its expression is reduced on microglia [38, 39]. Approximately 20% of CD45^-^ GFP^+^ cells in the retina expressed endothelial markers, CD31 and Tie-2, while 33% of them expressed microglial markers, CD45^dim^ CD11b^+^.

### Effect of diabetes on BM-derived inflammatory cells in the retina

**Fig 4:**
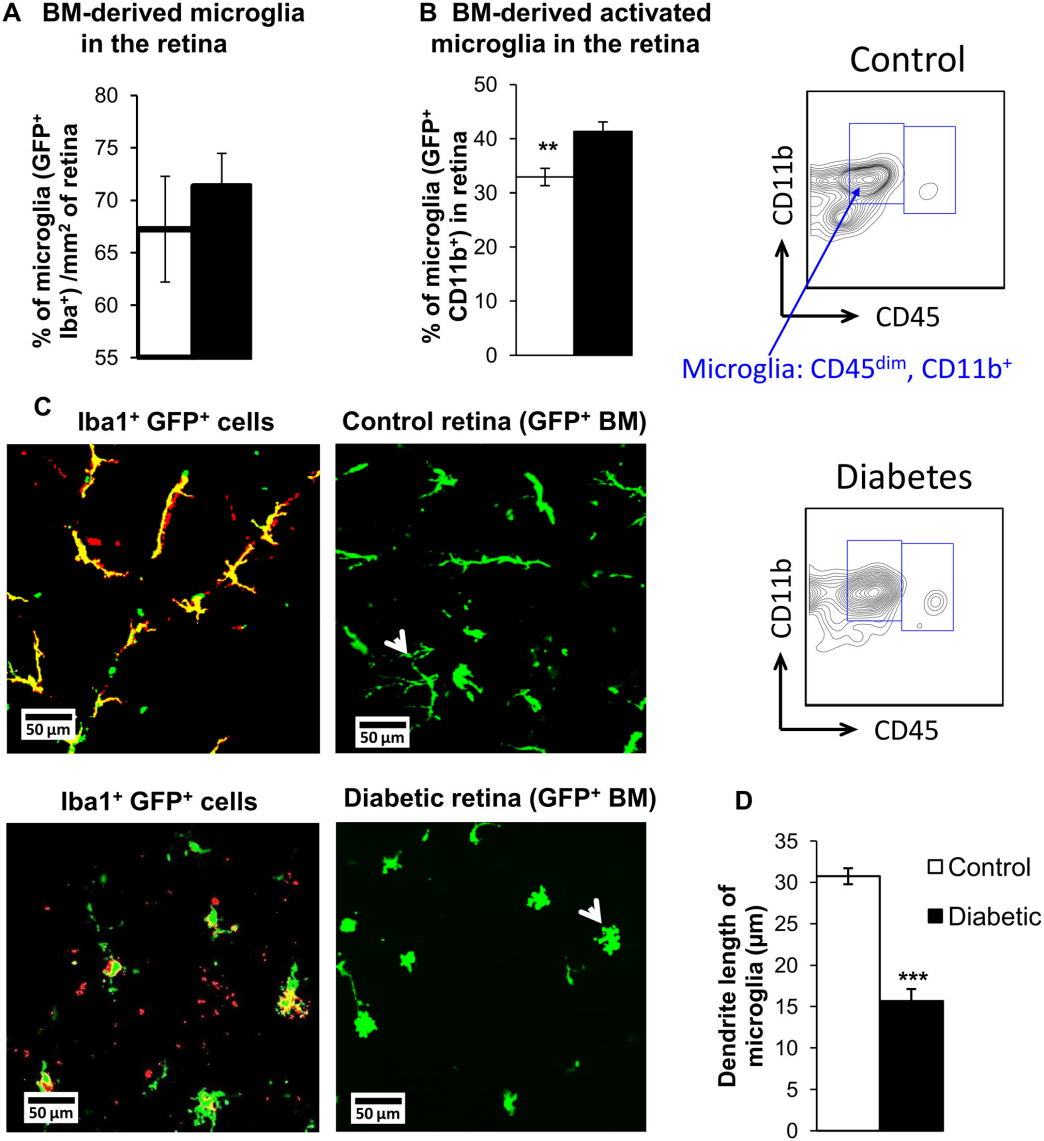
Diabetes alters BM-derived microglia in retina. (A) Percentage of BM-derived microglia per mm^2^ area of control or diabetic retina. N = 7–8. (B) GFP^+^ microglia (gated as GFP^+^ cells that are Thy1^-^, Ly6G^-^, Ly6C^-^, CD45^dim^ CD11b^+^ cells) are expressed as a percentage of total GFP^+^ retinal cells in control and diabetic retina of chimeric mice, and indicate increase in BM-derived microglia in diabetic mice. Representative flow charts of GFP^+^ microglia in the retina are shown. N = 3–5, ** p< 0.01. (C) Confocal images of retina isolated from control or diabetic GFP^+^ BM-transplanted mice. Microglial marker Iba-1^+^ staining (red) with GFP^+^ (green) cells, showing colocalization (yellow) in retina. Increased retraction of processes observed in BM-derived microglia in diabetic GFP^+^ chimeric mouse retina compared to ramified, resting phenotype of microglia in control retinas (white arrowheads). Scale bars are 50 μm. (D) Quantification of dendrite length of microglia in diabetic and control chimeric mouse retinas is shown. N = 4–5, *** p< 0.001.

To determine whether diabetes has an effect on infiltration of pro-inflammatory BM cells into the retina, we examined the retinas of chimeric mice 2 months after the induction of diabetes. We observed similar total numbers of GFP^+^ cells as well as GFP^+^ CD45^dim/-^ infiltrating diabetic and control retinas (Fig 3A and 3B). We then examined the effect of diabetes on infiltration of BM-derived microglia-like cells in the retina of chimeric mice. Using immunohistochemistry to stain retinas with the pan-microglial marker Iba-1, we observed no significant difference between control and diabetic mice in infiltration of total BM-derived microglia-like cells into the retina (Fig 4A). Next, we used flow cytometry to select for GFP^+^ CD45^dim^ CD11b^+^ cells in diabetic and control retinas, since high CD11b expression indicates increased microglial activation [40, 41]. Our studies revealed that 41% of GFP^+^ cells in diabetic retinas expressed activated microglial markers compared to 33% in control retinas (Fig 4B). The significant increase in percentage of CD11b^+^ GFP^+^ cells in diabetic retinas indicates increased infiltration and/or differentiation of activated microglia-like cells from BM to the diabetic retina. To further explore how diabetes affects activation of BM-derived pro-inflammatory cells, we examined phenotype of the microglia-like cells in flat-mounted retinas. In the control mouse retinas, most of the BM-derived microglia-like cells (Iba-1^+^ GFP^+^) displayed a resting phenotype characterized by their highly ramified morphology. However, in the diabetic retina, these BM-derived cells displayed a more amoeboid morphology with marked retraction of their dendrites, indicating microglial activation. (Fig 4C and 4D; white arrowheads).

In addition to microglia, early diabetes is associated with activation of the Müller glia, which are important contributors to retinal inflammation [42]. We studied the effect of diabetes on infiltration of BM-derived Müller cells in the retina. From immunohistochemical staining of flat-mounted retinas and retinal sections, we observed that 10% of BM-derived cells in the retina expressed markers of Müller cells (glutamine synthetase) (Fig 1F). In the diabetic retina, the percentages of BM-derived cells expressing markers of Müller cells did not change.

### Diabetes increases BM-derived monocytes in circulation

**Fig 5:**
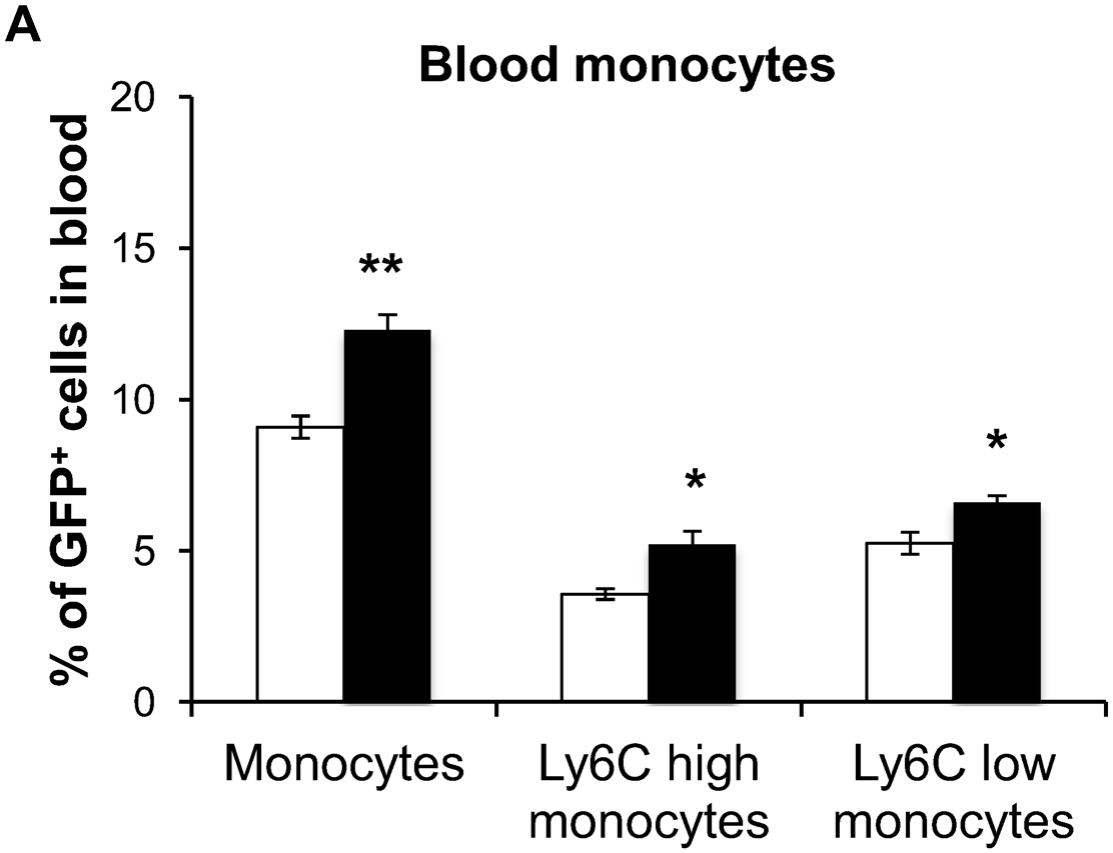
Effect of diabetes on BM-derived monocytes. GFP^+^ monocytes (CD11b^+^ Ly6C^+^, CD11b^+^ Ly6C^hi^ and CD11b^+^ Ly6C^lo^) are expressed as a percentage of total GFP^+^ cells in blood. N = 4–5, ** p< 0.01 and * p<0.05.

Next, we examined whether this increase in pro-inflammatory microglia-like cells is reflected in the numbers of the circulating progenitors of BM-derived microglia-like cells in control and diabetic mice. After 2 months of diabetes, we observed a significant increase in CD11b^+^ GFP^+^ circulating monocytes with surges of Ly6C^hi^ as well as Ly6C^lo^ monocyte populations in the blood of diabetic mice (Fig 5).

### Diabetes alters immune responses of BM-derived DC and splenocytes

**Fig 6:**
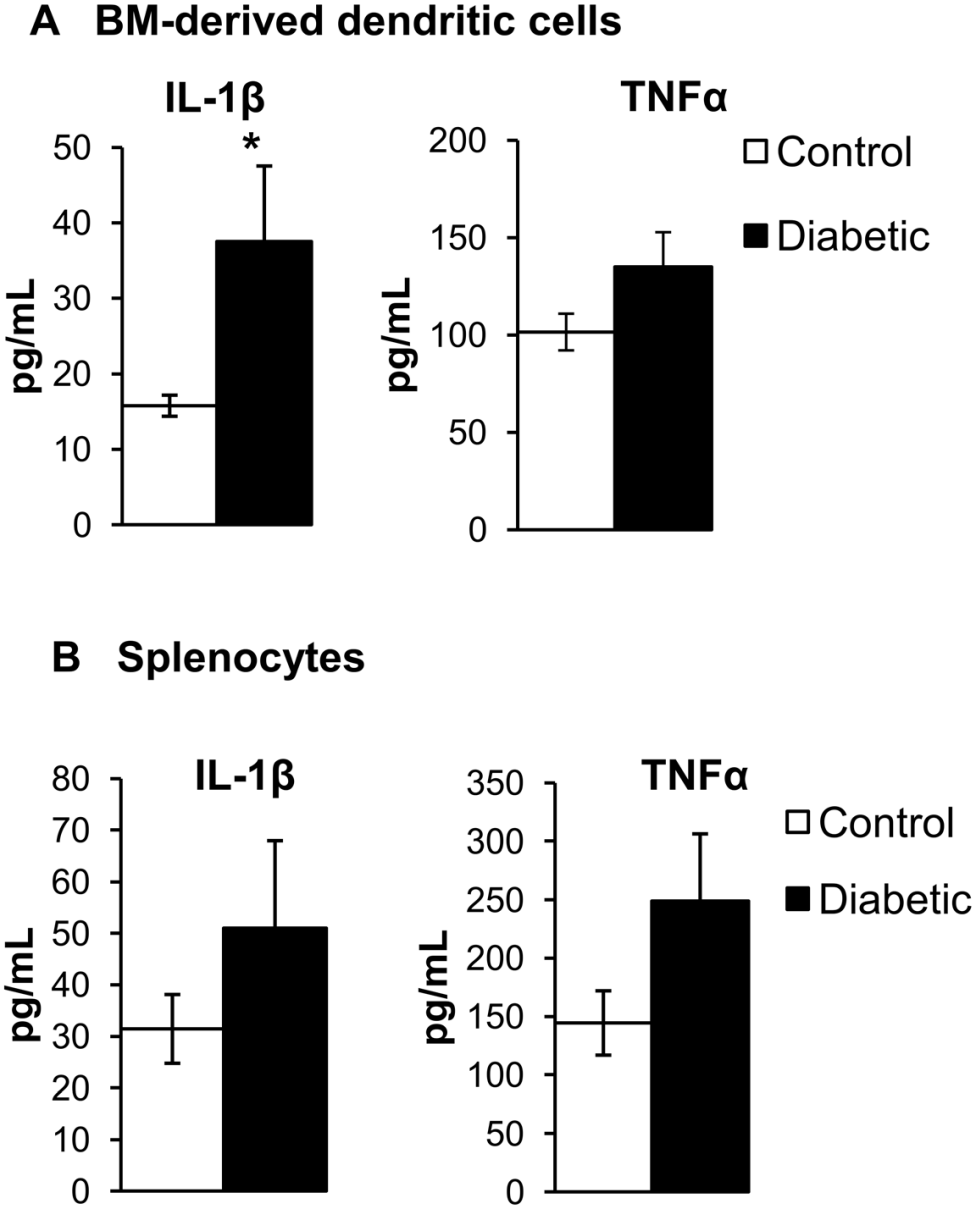
Diabetes alters response of BM-derived cells and splenocytes to LPS stimulation. Increased secretion of cytokines IL-1β and TNF-α in (A) BM-derived dendritic cell-enriched population (B) splenocytes stimulated with LPS. N = 4–5, * p< 0.05.

*In vitro* stimulation of cultured cells by lipopolysaccharide (LPS) is a widely used assay to study the ability of immune cells to become activated. For BM-derived DC and splenocytes, immune activation levels can be detected by changes in cytokine production, higher expression of costimulatory molecule or the production of inflammatory mediators [26]. To determine whether diabetes affects activation of BM-derived dendritic cells (DC) and splenocytes, we analyzed cytokine secretion by diabetic and control cell populations in response to LPS. We demonstrated that increased levels of pro-inflammatory cytokines, IL-1β and TNF-α were secreted by BM-derived DC and splenocytes derived from diabetic mice (Fig 6), indicating that diabetes may lead to aberrant activation of BM and splenic derived immune cells.

### Effect of diabetes on BM-derived microvascular cells in the retina

**Fig 7:**
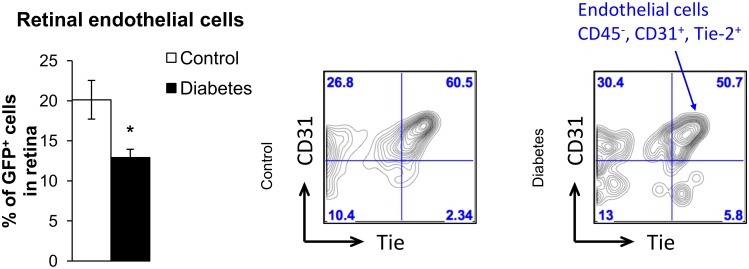
Diabetes reduces number of BM-derived endothelial cells in retina. GFP^+^ endothelial cells (gated as CD45^-^ Tie-2^+^ CD31^+^ cells) are expressed as a percentage of total GFP^+^ retinal cells, and indicate a significant decrease in BM-derived endothelial cells in diabetic mice. Representative flow charts of GFP^+^ endothelial cells in the retina are shown. N = 4–5.

To determine whether diabetes has an effect on infiltration of vascular reparative BM cells into the retina, we examined the retinas of chimeric mice after 2 months of diabetes. We observed that only 13% of GFP^+^ cells expressed endothelial cell markers compared to 20% in control retinas (Fig 7), indicating that infiltration and/or differentiation of BM-derived progenitor cells into retinal endothelial cells is deficient in the diabetic retina.

Pericytes are microvascular mural cells providing support and stability to the retinal vasculature. Pericyte loss and microaneurysm formation are characteristic features of diabetic retinopathy [43]. We studied the effect of diabetes on infiltration of BM-derived pericytes in the retina. From immunohistochemical staining of flat-mounted retinas and retinal sections, we observed that 10% of BM-derived cells in the retina expressed markers of pericytes (PDGF-Rβ) (Fig 1F). In the diabetic retina, the percentages of BM-derived cells expressing the pericyte marker did not change.

### Diabetes impairs release of BM-derived vascular reparative cells

**Fig 8:**
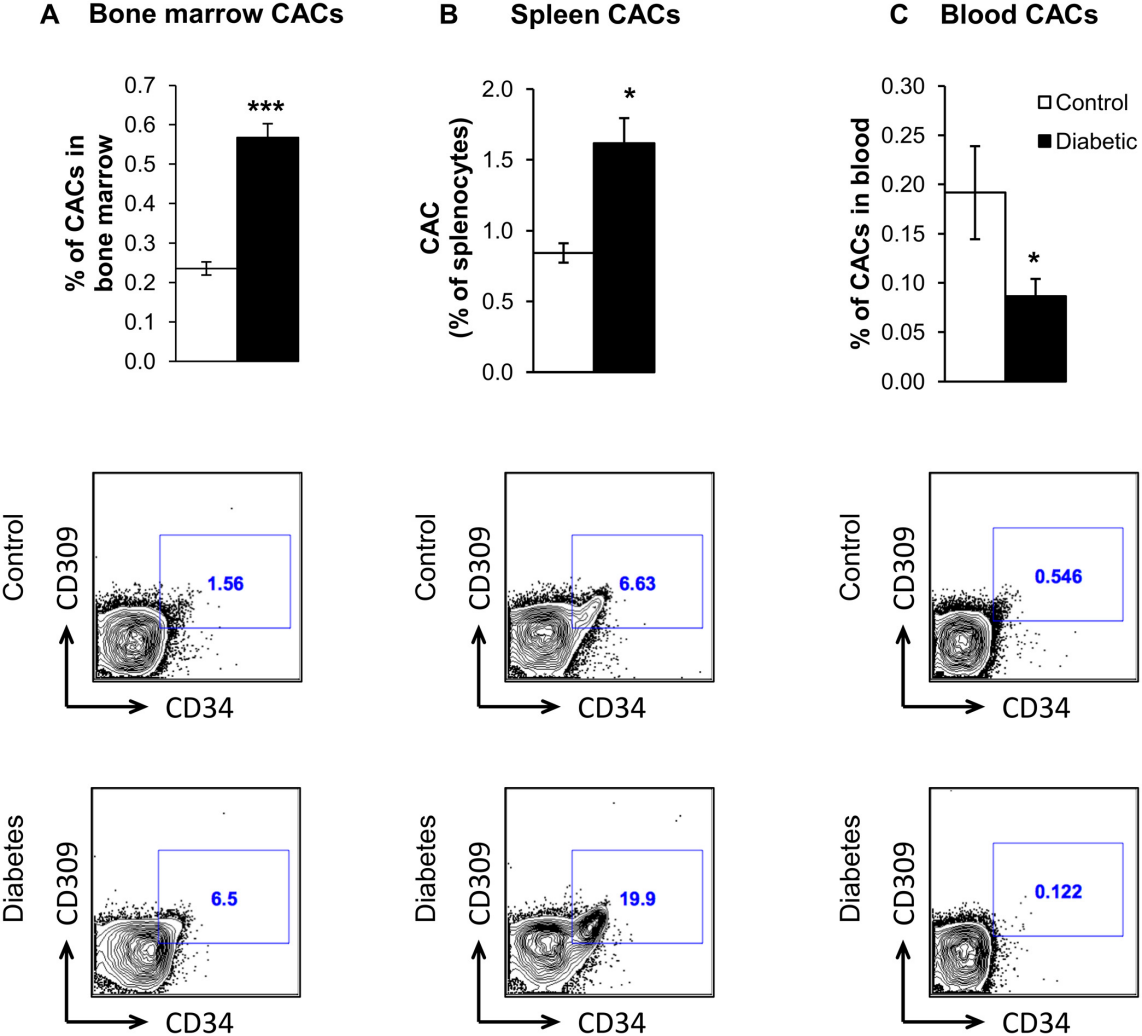
Diabetes alters release of vascular reparative cells into circulation. (A) CACs as a percentage of total bone marrow cells, *** p<0.001 (B) CACs as a percentage of total splenocytes. (C) CACs as a percentage of total blood cells. CACs are gated as Lin^-^ CD34^+^ CD309^+^ cells. Representative flow charts of GFP^+^ CACs in control and diabetic BM, spleen and blood are shown below. * p<0.05, N = 4–8.

To study the effect of diabetes on the release of vascular reparative cells, we tracked the movement of BM-derived CACs in chimeric mice. CACs were identified by flow cytometry as CD45^dim/+^ cells that do not express lineage markers but express CD34 and CD309 [44]. After 2 months of diabetes, we observed impaired release of CACs, shown as a significantly higher number of these cells trapped in the BM and spleen of diabetic mice compared to controls (Fig 8A and 8B). Significantly lower numbers of BM-derived CACs were observed in the blood of diabetic mice, as compared to controls (Fig 8C).

### CAC homing capability to BM is impaired in diabetes

**Fig 9:**
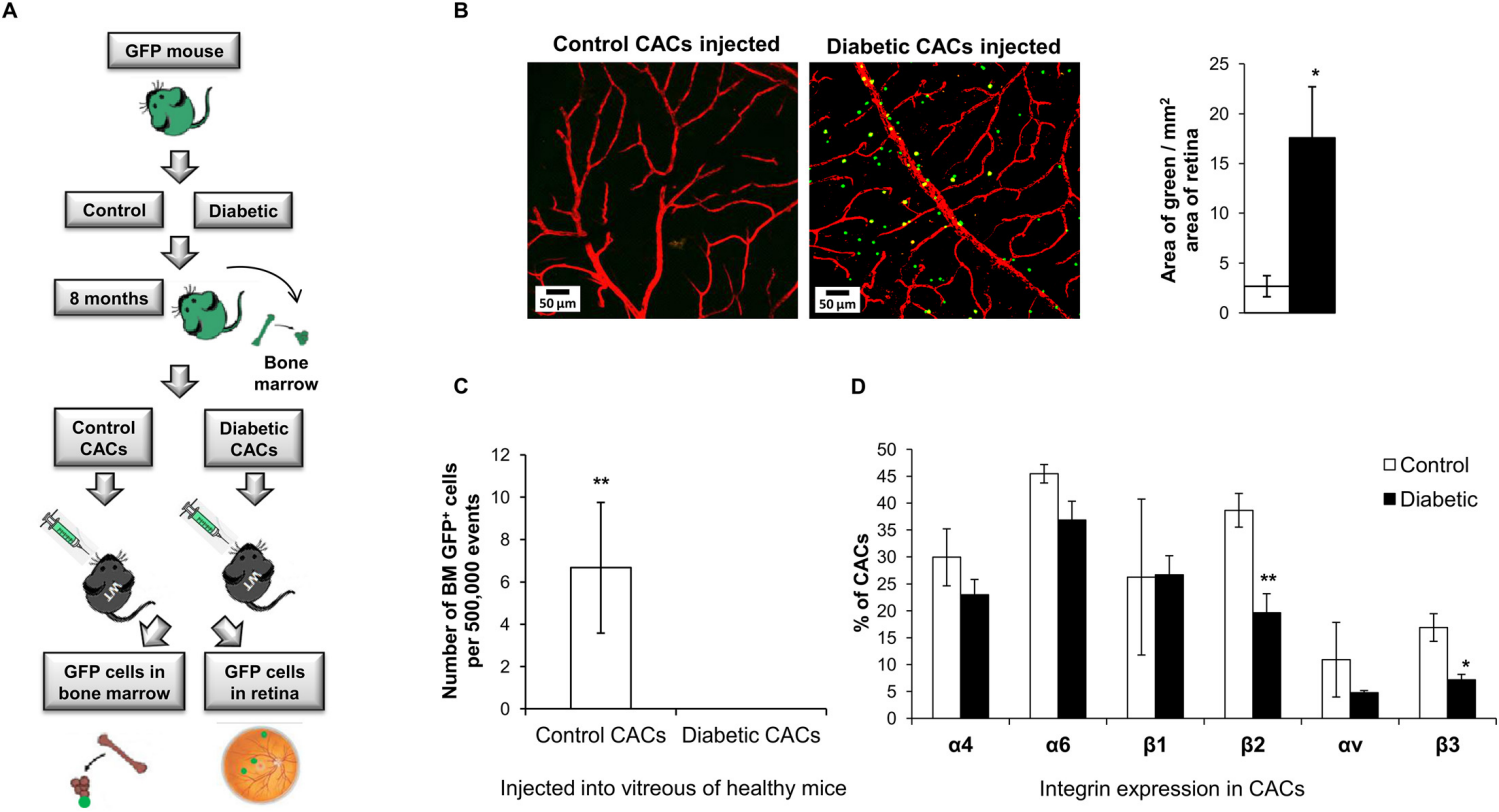
Diabetes alters homing efficiency of CACs. (A) Scheme for testing homing efficiency of diabetic CACs to the BM. Male C57BL/6-Tg(CAG-EGFP) mice were made diabetic by STZ injections. After 8 months of diabetes, 10,000 CACs were isolated from BM of diabetic and control GFP^+^ mice, and injected into the vitreous of healthy mice. Seven days post injection the BM and retinas were collected and analyzed by flow cytometry and confocal microscopy for presence of GFP^+^ CACs. (B) Control or diabetic GFP^+^ CACs (green) injected into the vitreous of mice with healthy retinal vasculature stained red using anti-collagen IV antibody. Quantitation of area of green CACs observed in confocal retinal images is shown. Scale bars are 50 μm, N = 10–12. (C) Quantification of GFP^+^ cells from bone marrow of tibia and femurs of recipient mice shows homing of diabetic GFP^+^ CACs to the recipient bone marrow was significantly lower compared to control GFP^+^ CACs. N = 4–6. (D) Diabetes significantly alters expression of integrins β2 and β3 on diabetic CACs in blood. N = 3, * p< 0.05, ** p<0.01.

In order to study the recirculation of CACs from the retina to the BM niche, we injected GFP^+^ CACs in the vitreous of control mice with healthy retinal vasculature. Seven days post injection, we examined the retina as well as BM for presence of GFP^+^ cells (Fig 9A). With injection of control GFP^+^ CACs, very few remained trapped in the vitreous, while GFP^+^ cells were detected in the BM, demonstrating that the injected CACs are capable of homing to their BM niche. However, with injection of diabetic GFP^+^ CACs, we observed a significant number of CACs trapped in the vitreo-retinal space, while no GFP^+^ cells were detected in the bone marrow (Fig 9B and 9C), implying impaired migration and homing efficiency of diabetic CACs.

To determine whether diabetes has an effect on integrins that regulate the process of CAC homing, we examined the expression of integrins α4, α6, β1, β2, αv and β3 on CACs isolated from the blood of control and diabetic mice. We observed significantly decreased expression of integrins β2 and β3, as well as a trend towards decreased expression of α4, α6 and αv integrins on diabetic CACs (Fig 9D). These data demonstrate that diabetes alters expression of integrins on the surface of CACs in peripheral blood, indicating that these adhesion molecules may be involved in dysregulation of CAC migration and homing efficiency associated with diabetes.

## Discussion

Diabetes affects the entire neurovascular unit of the retina, leading to chronic low-grade inflammation [45], gradual neurodegeneration [46], loss of capillary components such as pericytes and endothelial cells [47], causing acellular capillaries formation [48] and increased vascular permeability [49, 50]. The resulting loss of cellular support leads to microaneurysms, leakage of lipid exudates due to increased permeability, capillary non-perfusion, and subsequent ischemia and hypoxia of retinal tissue [50]. Many factors contribute to the pathophysiology of DR, such as accumulation of advanced glycation end-products (AGEs), inflammation, chronic oxidative stress and vascular as well as neuronal dysfunction [2, 51–54]. Diminished insulin signaling in retinal neurons may lead to neurodegeneration, which may further contribute to breakdown of the blood-retinal barrier in DR [46].

More recently, another mechanism, inadequate repair by deficient CAC in diabetes has been implicated in the pathogenesis of DR [7, 13, 30, 55]. The combination of retinal cell damage, pro-inflammatory changes and failed attempts by BM-derived CACs to repair injured retinal capillaries eventually result in progression to clinically significant DR. With significant contribution of BM-derived cells to retinal pathology in DR, it is important to understand the effect of diabetes on BM-derived cells contributing to retinal inflammation, as well as the cells promoting retinal vascular repair.

In agreement with previous studies, our data demonstrate that BM-derived cells infiltrating the retina differentiate into various cell types such as pericytes, endothelial cells, Müller cells and microglia (Figs 1 and 2) but not astrocytes or retinal neurons [33, 35]. Diabetes did not affect the numbers or activation status of BM-derived Müller cells and pericytes, indicating that these cells may be recruited from BM for normal maintenance functions in both control and diabetic retina [56]. However, diabetes significantly altered the numbers of BM-derived activated microglia-like cells as well as endothelial cells in the retina, suggesting that these BM-derived cell types may be involved in the pathogenesis of DR (Figs 4B and 7).

Diabetes is known to activate monocytes, which play an important part in promoting a pro-inflammatory environment in the retina [9, 10]. Circulating monocytes are classified as Ly6C^hi^ or Ly6C^lo^, depending on their expression levels of Ly6C on the cell surface. Ly6C^lo^ monocytes perform surveillance functions and resolve inflammation, while Ly6C^hi^ monocytes are characterized as reactive cells that can be actively recruited into diabetic retina, contributing to the observed pathology [30, 57]. We and others have previously demonstrated a shift in the profile of BM cells and circulating BM-derived cells towards myeloid cells, contributing to diabetes-associated inflammation [12, 30, 51, 58]. Indeed, reactive (Ly6C ^hi^) as well as the patrolling (Ly6C ^lo^) monocyte subsets of this BM-derived population (GFP^+^ CD11b^+^) were found to be increased in the blood of diabetic chimeric mice (Fig 5).

Under normal conditions, retinal microglia survey the retinal microenvironment and help maintain homeostasis. Resident microglia, along with astrocytes are also believed to play a vital role in formation and maintenance of the retinal vasculature [59]. However, during inflammation these microglia become activated and produce cytokines, contributing to vascular and neural damage in the diabetic retina [60]. Circulating monocytes can infiltrate the diabetic retina, assume a microglia-like phenotype and contribute to retinal inflammation by secreting pro-inflammatory cytokines and further activating resident glial cells in the retina [38, 61, 62]. In our study, we demonstrate increased infiltration of BM-derived pro-inflammatory cell types expressing microglial activation marker CD11b in the diabetic retina (Fig 4B). Interestingly, there was no change in total BM-derived microglial population in these retinas as demonstrated using a pan-microglial marker Iba-1 (Fig 4A). These data indicate that the BM microenvironment in diabetes induces a shift in hematopoiesis with generation of more pro-inflammatory monocytes released from BM into circulation, leading to accumulation of inflammatory microglia-like cells in the diabetic retina. Since the numbers of BM-derived activated microglia-like cells are selectively increased in the diabetic retina with no change in total microglia numbers, the beneficial role of resting microglia in vascular maintenance and surveillance is likely to be diminished in diabetes.

During embryonic development, resident tissue microglia are known to develop from a yolk sac and do not have myeloid origin [63]. Several studies demonstrated that in normal tissue, microglial regeneration occurs from tissue-specific microglial progenitors [64]. However, microglial origin could be shifted towards BM-derived myeloid cells in ageing, inflammation or tissue damage [38, 62, 65]. In experimental models of DR, microglial activation is usually identified by morphological changes involving retraction of their highly ramified processes and appearance of an amoeboid shape with thicker dendrites and larger cell bodies [42, 65]. We observed Iba^+^ GFP^+^ cells in control and diabetic retinas, however Iba^+^ GFP^+^ cells in control retinas had branched resting phenotype compared to clearly activated amoeboid phenotype in diabetic retinas, demonstrating for the first time that diabetes promotes activation of BM-derived microglia-like cells in the retina (Fig 4C and 4D).

The BM serves as a niche for hematopoietic stem cells which, apart from differentiating into lymphoid and myeloid progenitors, are also believed to give rise to CACs, a population of cells that circulates in the bloodstream with the ability to migrate to the site of endothelial injury and mediate repair of damaged blood vessels. These BM progenitors may migrate to the spleen, which serves as a reservoir for CACs, inflammatory monocytes and lymphocytes [21–23]. We and others have previously shown that diabetes affects mobilization of CACs into systemic circulation [7, 28]. Studies by us and others also indicate that CACs isolated from diabetic patients are not effective in vascular regeneration due to their impaired migration and proliferation abilities [7, 11, 13]. In this study, we show that release of CACs from BM and spleen into circulation is impaired in a mouse model of diabetes (Fig 8A–8C).

Further, we have demonstrated that 20% of BM-derived cells that infiltrate the retina express endothelial cell markers such as collagen IV, Tie2, PECAM-1 (Fig 7). A previous study by Grant *et al* also demonstrated that adult hematopoietic stem cells are capable of migrating to the retina and differentiating into endothelial cells [66]. However, in the diabetic retina, we observed a significant reduction in the percentage of BM-derived cells expressing endothelial markers, reinforcing the notion that migration and/or differentiation of BM-derived progenitor cells into retinal endothelial cells is deficient in diabetes (Fig 7).

CACs arising from the BM, spleen and other niches normally circulate in the bloodstream and home to areas of endothelial injury to mediate vascular repair [66]. In a healthy animal, chemokine gradients such as SDF-1 and up-regulation of CXCR-4 receptors on CACs play crucial roles in regulating release, surveillance and homing of reparative CACs to sites of retinal vascular injury which is disturbed in diabetes, as we have previously described [7, 17, 18]. In order to maintain their population, these cells migrate from various tissues back to their niches, governed by expression of integrins and chemokine gradients [14, 17, 20]. In this study we demonstrate for the first time, impairment in homing of diabetic CACs from healthy retina back to their niches, using BM niche as an example of a major reservoir for CACs. To observe the effect of diabetes on the *in vivo* migration and homing capacity of CACs, we injected GFP^+^ CACs isolated from 8-month diabetic and age-matched control mice, into the vitreous of wild type mice with healthy retinal vasculature. Seven days post injection, the retina and BM were collected and analyzed for presence of residual GFP^+^ CACs (Fig 9A). As there was no retinal damage in the recipient mice, normal control CACs did not remain in the vitreo-retinal space and migrated out into the blood stream as expected (Fig 9B, left panel). In contrast to the control CACs, a significant number of diabetic GFP^+^ CACs remained in the vitreo-retinal space (Fig 9B, right panel) unable to migrate.

Next, we examined the BM of the recipient mice for presence of GFP^+^ cells. It is important to note that only 10,000 GFP^+^ CACs were injected into the vitreous of WT non-GFP^+^ mice. The only source of GFP^+^ cells in the BM of animals would be these CACs that were injected into the vitreous. Even if all the injected GFP^+^ cells homed to niches in the bone marrow, we would expect to see only 70–100 GFP^+^ cells in 500,000 bone marrow cells. However, CACs have also been shown to migrate to other niches such as spleen, liver and peripheral blood, and may participate in vascular maintenance in other parts of the body [28]. Thus 7 ± 3 cells per 500,000 cells observed per stem cell niche is within the range that can be expected in this experimental set-up (Fig 9C). In contrast, the impairment in migration and homing efficiency of diabetic CACs is apparent in the observed absence of diabetic GFP^+^ CACs circulating and homing back to the BM niche (Fig 9C).

Regulation of CAC homing involves chemokine gradients and expression of receptors and adhesion molecules on CACs. Previously, we and others demonstrated that chemokines regulating CAC release and mobilization such as SDF-1, VEGF and MCP-1 are under stringent circadian control, which is altered in diabetes [7, 67]. As this study was not designed to address circadian changes, we did not analyze the potential contribution of chemokine gradients to the observed changes in the release of BM progenitor cells in diabetes. Adhesion molecules expressed on the surface of CACs, called integrins are also important determinants of CAC homing. Intgrins α6β1 and αvβ3 regulate homing and adhesion of CACs to vasculature, β2 integrins are major regulators of transendothelial migration and integrin α4β1 is an important regulator of CAC retention in the BM by binding to VCAM1 on endothelial cells [19, 20, 68]. In our study, we demonstrate for the first time that diabetes results in significantly reduced expression of β2 and β3 integrins, as well as a trend towards decreased expression of α4, α6 and αv integrins on CACs (Fig 9D). This may in turn affect integrin-mediated interactions of CACs with the vessel wall, contributing to the observed impairment in migration and homing efficiency of diabetic CACs.

The reduction in the number and homing efficiency of CACs in diabetes was accompanied by aberrant activation of splenocytes and BM-derived dendritic cells upon LPS stimulation. Immature DC can be activated by LPS and secrete cytokines that influence the leukocyte immune response [24–26]. Lymphocytes, dendritic cells and monocytes stored in the spleen may also contribute to inflammation in response to injury [23, 24]. Here we show that LPS stimulation leads to increased secretion of pro-inflammatory cytokines such as IL-1β and TNF-α by splenocytes and BM-derived dendritic cells enriched from diabetic mice, indicating that these immune cells may also contribute to inflammation in diabetes (Fig 6).

Our study is in agreement with the main findings of a previous study by Li *et al*, on the role of pro-inflammatory marrow-derived cells in DR. However, there are important differences in the design of the two studies. In the Li *et al*. study diabetes was induced two weeks before BM transplantation [33]. As one of the goals of our study was to evaluate migration and homing of the progenitor cells between different niches, we wanted to separate the effects of irradiation from the effects of diabetes and allowed for stable bone marrow engraftment (4–5 months) before induction of diabetes. This study design with BM transplantation before induction of diabetes assures that there are no effects of diabetes on homing efficiency of transplanted BM cells and subsequent re-population of stem cells in the BM niche.

In conclusion, this study identified a significant shift from reparative to pro-inflammatory BM-derived cells in the retina in diabetes. The reparative BM-derived CACs had decreased numbers in circulation, as well as deficient migratory and homing capacity in diabetes. In contrast, diabetes induced higher numbers in circulation, as well as retinal infiltration of pro-inflammatory myeloid cells giving rise to activated microglia-like population in diabetic retina. Control of BM-derived cell populations with normalization of the reparative/pro-inflammatory cells balance could represent a viable cell therapy option to enhance available DR treatments.
